# Supplementary material for: Genetic composition of queen conch (Lobatus gigas) population on Pedro Bank, Jamaica and its use in fisheries management
Source: PLoS One. 2021 Apr 5;16(4):e0245703. doi: 10.1371/journal.pone.0245703 (PMC8021194; doi:10.1371/journal.pone.0245703)
Supplement: S1 Table — (DOCX) [file pone.0245703.s001.docx]

**S1 Table. G-statistics calculated for Pedro Bank.**

| **Locus** | **Fst** | **Gis** | **Gst** | **G'stN** | **G'stH** | **G''st** | **Dest** | **P (Gst)** |
| --- | --- | --- | --- | --- | --- | --- | --- | --- |
| **ConchPR11** | 0.014 | -0.016 | 0.005 | 0.006 | 0.106 | 0.107 | 0.101 | 0.007 |
| **Sgig1** | 0.015 | 0.048 | 0.005 | 0.006 | 0.038 | 0.039 | 0.033 | 0.057 |
| **Conch17** | 0.014 | -0.040 | 0.005 | 0.006 | 0.046 | 0.047 | 0.041 | 0.033 |
| **Conch29** | 0.007 | -0.029 | -0.002 | -0.003 | -0.016 | -0.016 | -0.013 | 0.796 |
| **ConchPR1** | 0.035 | -0.042 | 0.026 | 0.032 | 0.324 | 0.328 | 0.306 | 0.001 |
| **Sgig2** | 0.006 | -0.068 | -0.003 | -0.004 | -0.011 | -0.012 | -0.008 | 0.773 |
| **Conch21** | 0.007 | -0.140 | -0.001 | -0.002 | -0.010 | -0.011 | -0.009 | 0.679 |
| **Conch23** | 0.046 | 0.053 | 0.036 | 0.044 | 0.086 | 0.094 | 0.051 | 0.001 |
| **Sgig6** | 0.008 | -0.046 | -0.002 | -0.002 | -0.005 | -0.006 | -0.004 | 0.622 |
| **Tot** | 0.016 | -0.033 | 0.007 | 0.009 | 0.039 | 0.041 | 0.033 | 0.001 |
| **SE** | 0.004 | 0.019 | 0.004 | 0.005 | 0.023 | 0.024 | 0.019 |  |
| **CIU** | 0.010 | -0.067 | 0.001 | 0.001 | 0.004 | 0.004 | 0.003 |  |
| **CIL** | 0.024 | 0.001 | 0.014 | 0.018 | 0.083 | 0.086 | 0.072 |  |

Summary of G-statistics and related Fixation index by locus as calculated over all populations. Fst: Fixation Index, Gis: Inbreeding coefficient within individuals, adjusted for bias, Gst: Analog of Fst, adjusted for bias, G’stN: Nei’s standardized Gst, G’stH: Hedrick’s standardized Gst, G”st: Hedrick’s standardized Gst further corrected for bias when number of populations is small, Dest: Jost’s estimate of differentiation, P: probability Gst.
