# Supplementary material for: Genetic composition of queen conch (Lobatus gigas) population on Pedro Bank, Jamaica and its use in fisheries management
Source: PLoS One. 2021 Apr 5;16(4):e0245703. doi: 10.1371/journal.pone.0245703 (PMC8021194; doi:10.1371/journal.pone.0245703)

**S1 Appendix. Scatterplot of principal component analysis showing five populations of *Lobatus gigas*, across Pedro Bank.**


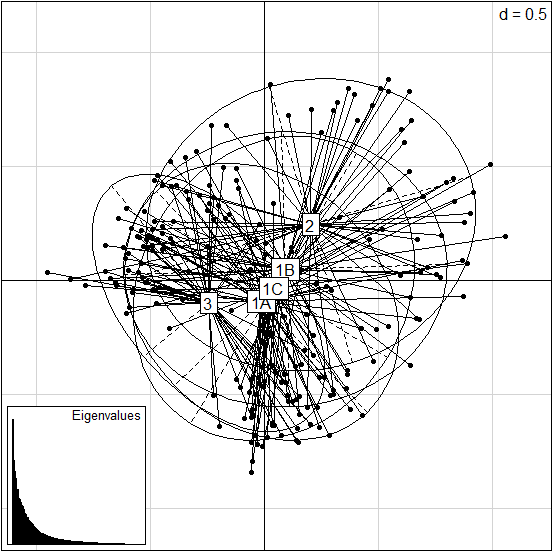

Supplement: S1 Appendix — (DOCX) [file pone.0245703.s002.docx]
