# Supplementary material for: Genetic composition of queen conch (Lobatus gigas) population on Pedro Bank, Jamaica and its use in fisheries management
Source: PLoS One. 2021 Apr 5;16(4):e0245703. doi: 10.1371/journal.pone.0245703 (PMC8021194; doi:10.1371/journal.pone.0245703)
Supplement: S2 Appendix — (DOCX) [file pone.0245703.s003.docx]

# S2 Appendix. Population Assignment Outcomes for 5 groups of individuals sampled on Pedro Bank to ‘Self’ or ‘Other’ Population

| **Pop** | **Self Pop** | **Other Pop** |
| --- | --- | --- |
| 1A | 28 | 18 |
| 1B | 21 | 19 |
| 1C | 25 | 17 |
| 2 | 26 | 8 |
| 3 | 42 | 4 |
| **Total** | 142 | 66 |
| **Percent** | 68% | 32% |
