# Supplementary material for: Genetic composition of queen conch (Lobatus gigas) population on Pedro Bank, Jamaica and its use in fisheries management
Source: PLoS One. 2021 Apr 5;16(4):e0245703. doi: 10.1371/journal.pone.0245703 (PMC8021194; doi:10.1371/journal.pone.0245703)
Supplement: S3 Appendix — (DOCX) [file pone.0245703.s004.docx]

# S3 Appendix. Bayesian clustering analysis of samples from the 2015 Queen Conch abundance survey on Pedro Bank performed in STRUCTURE.

| K | Ln P(D) | Var [Ln P(D)] |
| --- | --- | --- |
| 2 | -5150.7 | 246.7 |
| 3 | -5109.4 | 389.4 |
| 4 | -5462.6 | 1166 |
| 5 | -5769.2 | 1910 |

**S3 Appendix A. Estimates of log probability under assumptions K = 2-5**

**S3 Appendix B. Number of homogeneous populations (K) and their posterior probabilities [Ln P(D)].**


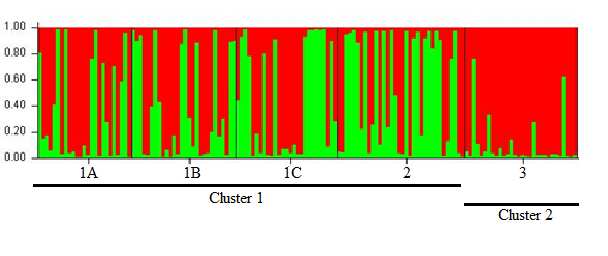


**S3 Appendix C. Bar plot indicating 2 clusters which represents the most reliable populations from the samples analysed**.
